# Regulation of plasmalogen metabolism and traffic in mammals: The fog begins to lift

**DOI:** 10.3389/fcell.2022.946393

**Published:** 2022-08-31

**Authors:** Fabian Dorninger, Ernst R. Werner, Johannes Berger, Katrin Watschinger

**Affiliations:** ^1^ Department of Pathobiology of the Nervous System, Center for Brain Research, Medical University of Vienna, Vienna, Austria; ^2^ Institute of Biological Chemistry, Biocenter, Medical University of Innsbruck, Innsbruck, Austria

**Keywords:** phospholipid, precursor treatment, lipid traffic, plasmalogen degradation, plasmalogen remodeling, plasmalogen biosynthesis, ether lipids, lipid metabolism

## Abstract

Due to their unique chemical structure, plasmalogens do not only exhibit distinct biophysical and biochemical features, but require specialized pathways of biosynthesis and metabolization. Recently, major advances have been made in our understanding of these processes, for example by the attribution of the gene encoding the enzyme, which catalyzes the final desaturation step in plasmalogen biosynthesis, or by the identification of cytochrome C as plasmalogenase, which allows for the degradation of plasmalogens. Also, models have been presented that plausibly explain the maintenance of adequate cellular levels of plasmalogens. However, despite the progress, many aspects around the questions of how plasmalogen metabolism is regulated and how plasmalogens are distributed among organs and tissues in more complex organisms like mammals, remain unresolved. Here, we summarize and interpret current evidence on the regulation of the enzymes involved in plasmalogen biosynthesis and degradation as well as the turnover of plasmalogens. Finally, we focus on plasmalogen traffic across the mammalian body – a topic of major importance, when considering plasmalogen replacement therapies in human disorders, where deficiencies in these lipids have been reported. These involve not only inborn errors in plasmalogen metabolism, but also more common diseases including Alzheimer’s disease and neurodevelopmental disorders.

## 1 Introduction

The discovery of plasmalogens in 1924 as an unknown, aldehyde-releasing substance present in plasma (plasmal-ogen) was an accidental observation owed to Robert Feulgen’s group preferring to spend a hot summer day off in the woods and on the next day - tired of the day before - forgetting to put the HgCl_2_-stained tissue slices into fixative and acid before staining with fuchsin-sulfurous acid (Debuch and Seng, 1972). In contrast to obtaining the expected intense nuclear purple-staining of DNA-derived aldehydes forming a Schiff base with the colorless acidic fuchsin solution - a nuclear staining method established in the Feulgen lab - they found fuchsin-staining also in the cellular plasma. Further investigations then revealed that these aldehydes derived from lipids. Nowadays, it has emerged, that these accidentally identified lipids are important and intriguing because of their involvement in different disease spectra and their distinct biophysical properties distinguishing them from other membrane lipids.

Plasmalogens, which are also called plasmenyl lipids, belong to the group of ether phospholipids (or short only ether lipids). They are discriminated from their metabolic precursors, the plasmanyl lipids, by a cis vinyl ether double bond (Norton et al., 1962; Warner and Lands, 1963). If both plasmanyl and plasmenyl lipids are absent, like in the peroxisomal disorders rhizomelic chondrodysplasia punctata (RCDP) and Zellweger spectrum disorders, but also in knockout mouse models with deletion of one of the initial, peroxisomal steps of plasmanyl/plasmenyl lipid biosynthesis, symptoms of affected individuals include impaired growth and neurological development, cataracts, and bone phenotypes with mouse models being generally milder than the respective human disease (Berger et al., 2016), where most patients die during childhood (Duker et al., 2020). So far, it is, however, not possible to clearly attribute symptom development to either missing plasmanyl lipids or plasmalogens, a line of research we are currently following. Decreased levels of plasmalogens have also been associated with smoking-related lung disease (Braverman and Moser, 2012) and were found in brains of Alzheimer’s (Kou et al., 2011) and Parkinson’s (Fabelo et al., 2011) disease patients. It is, however, still not clear whether loss of plasmalogens is causative in the etiology of these diseases or a consequence of them.

Plasmalogens are present in animals but are missing in plants and fungi (Goldfine, 2010). Knowledge on the genetics of plasmalogen biosynthesis was only very recently expanded with *Tmem189* (transmembrane protein of unknown function 189) having been shown to code for plasmanylethanolamine desaturase (PEDS1), the enzyme that introduces the crucial vinyl ether double bond into plasmanylethanolamines (PE[O]) in animals and thereby gives rise to the lipid subclass of plasmalogens (Gallego-García et al., 2019; Werner et al., 2020; Wainberg et al., 2021). Some bacteria, especially anaerobes, can also synthesize plasmalogens, however, they lost this capacity upon increase of oxygen in the atmosphere, possibly due to the sensitivity of plasmalogens to O_2_. Later in evolution, plasmalogen biosynthesis reappeared in bacteria and was introduced rapidly also in animals (Goldfine, 2010). A distinct metabolic pathway for plasmalogen biosynthesis in bacteria was recently discovered (Jackson et al., 2021).

About 20% of all phospholipids in a human body are plasmalogens, which carry almost exclusively ethanolamine or choline as head group (Braverman and Moser, 2012). Such high values suggest that they are not only storage molecules for inflammatory mediators and signaling precursors but that they are required for shaping the properties of biological membranes (Koivuniemi, 2017)**.** In this regard, Horrocks and Sharma stated in 1982 that plasmalogens are more loosely packed than their diacyl counterparts thereby increasing membrane fluidity, and that they differ in their surface potential from other phospholipids (Horrocks and Sharma, 1982). In 1984, the Paltauf group in Graz showed that ethanolamine plasmalogens (plasmenylethanolamine; PE[P]) are more likely to adopt the inverted hexagonal phase because they have lower lamellar gel to liquid-crystalline and lamellar to inverse-hexagonal phase transition temperatures compared to their alkylacyl- and diacyl-homologues (Lohner et al., 1984). Presence of the vinyl ether double bond decreases hydrophilicity and leads to a perpendicular orientation of the acyl side chain at *sn*-2 relative to the membrane surface (Lohner, 1996). This intrinsic drive to form inverted hexagonal structures as well as their reduced transition temperature between lamellar and non-lamellar phase also make plasmalogens important determinants in membrane fusion (Lohner, 1996). However, this was only shown for PE[P] and not for choline plasmalogens (plasmenylcholine; PC[P]), which do not form non-bilayer structures at temperatures around 37°C (Lohner, 1996). In contrast to Horrocks’ and Sharma’s observation, a membrane-rigidifying effect was attributed to plasmalogens by studying membranes of Zellweger patients and comparing them to healthy individuals (Hermetter et al., 1989). This finding was validated by atomistic molecular dynamics simulation studies showing higher lipid membrane condensation and thickness in membranes consisting purely of PE[P] (Rog and Koivuniemi, 2016). So far, it is not clear which plasmalogen content is needed in biological membranes to make a significant impact on membrane properties.

Very recently, ether lipids including plasmalogens were shown to have pro-ferroptotic traits (Aldrovandi and Conrad, 2020; Zou et al., 2020). Ferroptosis is elicited by lipid peroxidation (Stockwell et al., 2017). The current hypothesis regarding ether lipids is that the high abundance of polyunsaturated fatty acids (PUFA) at the *sn*-2 position makes them prone to oxidation due to their chemical properties and therefore these lipids promote this form of orchestrated cell death (Zou et al., 2020). Also two enzymes of ether lipid metabolism which are crucial for plasmalogen biosynthesis were shown to be important for evoking ferroptosis (Cui et al., 2021). The first is fatty acyl-CoA reductase 1 (FAR1), the rate-limiting enzyme responsible for providing the fatty alcohol needed for the formation of the ether bond in peroxisomes (Cheng and Russell, 2004) and which is regulated in a feedback loop by the cellular amount of plasmalogens (see chapter 3.1.1.1 *Peroxisomal steps* and chapter 3.1.2 *Regulation of plasmalogen biosynthesis*) (Honsho et al., 2010). The second enzyme implicated in ferroptosis is PEDS1, however, Zou and co-workers did not identify PEDS1 in their CRISPR-screen (Zou et al., 2020). Further research will determine the exact impact of PEDS1 on ferroptosis and whether an enzyme exists that specifically enriches PUFA at the *sn*-2 position of plasmalogens, by distinguishing between plasmenyl and plasmanyl ether lipids.

Based on previous excellent reviews on the topic (Lessig and Fuchs, 2009; Braverman and Moser, 2012; Honsho and Fujiki, 2017; Dean and Lodhi, 2018; Dorninger et al., 2020), in this review we give an update on the knowledge of plasmalogen metabolism in mammals with a particular focus on steady state levels in health and disease, on the regulation of biosynthesis and remodeling as well as of the different ways for degradation. Furthermore, we highlight recent developments on trafficking of these lipids from dietary intake or autonomous biosynthesis to the distribution across the various tissues.

## 2 Steady state levels of plasmalogens in health and disease

The results of quantitative plasmalogen analyses are snapshots of the amount of plasmalogens at the time of extraction. In some investigations, the data reflect a mixture of many different cell types providing an overview of the situation in an entire tissue at an individual time point. It has long been known that lipid turnover rates vary strongly with respect to lipid species and tissue. One classical example is myelin, where the lipid turnover rate is much lower than in other brain-derived cell membranes (cf. chapter 3.3.2 *Turnover of plasmalogens*). Within cell membranes, lipid composition can change rapidly in response to environmental changes or in response to pathogens. For instance, plasmalogen levels increase in macrophages after cytomegalovirus infection (Jean Beltran et al., 2018). However, different techniques of plasmalogen determination must be distinguished for correct interpretation of the lipid changes. For example, the quantification of dimethylacetals (DMA) provides the amount of plasmalogens irrespective of the head group and the *sn*-2 fatty acid, but differentiating between the *sn*-1 alkyl chains (e.g., the total amount of C16:0, C18:1, C18:0 and C20:0 DMA). Thus, the sum of the most abundant DMA species yields a broad overview of general plasmalogen changes, like those resulting from altered biosynthesis or degradation. In contrast, lipidome analysis can identify changes in individual plasmalogen species in spite of unchanged overall plasmalogen levels, for example due to remodeling (Koch et al., 2022). With this distinction in mind, for example the robust changes in individual plasmalogen species observed in macrophages 120 h post viral infection might be a result of remodeling of the *sn*-2 position rather than being caused by increased plasmalogen synthesis, as most common plasmalogen species remain unaltered (Jean Beltran et al., 2018). On the other hand, the abnormalities in brain tissue of patients with Alzheimer’s disease seem to represent a general reduction of total plasmalogen levels, as proven by different methods (Guan et al., 1999; Han et al., 2001; Grimm et al., 2011b; Kou et al., 2011). The inverse correlation between plasmalogen levels and pathology markers can be observed by both the sum of the most abundant DMA species (C16:0, C18:1, and C18:0) and common as well as uncommon individual plasmalogen species (Kou et al., 2011). It is known that in cell culture and in entire tissues compensatory adaptive lipid changes occur in response to the reduction or increase by exogenous supplementation of plasmalogens keeping the overall levels of ethanolamine phospholipids constant (Dorninger et al., 2015). Whether or not the same tight compensatory mechanisms also apply to lipids with other head groups is currently not known, presumably due to former difficulties in the identification of PC[P] species.

When considering the biological relevance of plasmalogen alterations, the head group is of crucial importance. In particular, the asymmetric distribution of different plasmalogen subclasses between the cytofacial and exofacial leaflets of the plasma membrane is not only essential for physical membrane properties, but possibly also for the ability of PLA2 to release PUFA from the *sn*-2 position. These considerations are particularly relevant given the involvement of these important fatty acids in signaling (Bazinet and Layé, 2014). It has been demonstrated by Fellmann and others that PE[P], like its diacyl counterpart phosphatidylethanolamine (PE), diffuses rapidly from the outer to the inner leaflet, whereas only less than 20% of PC[P] molecules, comparably to phosphatidylcholine (PC), reach the interior face of the membrane after 4 h. Thus, plasmalogens behave as the corresponding diacyl lipids in this respect (Fellmann et al., 1993). PE[P] has been suggested to accumulate in lipid rafts (also termed membrane rafts) (Pike et al., 2002), membrane microdomains enriched in cholesterol and sphingolipids, which compartmentalize cellular processes. However, confirmatory follow-up data on this issue are lacking and the concept of lipid rafts has been in constant flux over the years (Levental et al., 2020), thus clouding the potential role of plasmalogens within these membrane compartments. Also other membrane subdomains, caveolae and clathrin-coated pits, which are important for endo- and exocytotic processes, are dependent on plasmalogens as indicated by the fact that their morphology is altered upon ether lipid deficiency (Thai et al., 2001), which is in line with the fusogenic properties ascribed to plasmalogens (Glaser and Gross, 1995). Apart from their potential enrichment in lipid rafts, plasmalogens have an additional connection to cholesterol: the levels of plasmalogens have been reported to influence cholesterol biosynthesis by modulating the stability of one of the key enzymes in the pathway, squalene monooxygenase (Honsho and Fujiki, 2017; Honsho et al., 2019).

### 2.1 Plasmalogen levels in different tissues

The contribution of plasmalogens to the total amount of phospholipids differs strongly between mammalian tissues with the highest amount being present in the myelin sheath, where plasmalogens account for about 31%–37% of all phospholipids (Horrocks, 1972). In brain white matter, which contains large amounts of myelin, PE[P] makes up about 85 mol% of all ethanolamine phospholipid species. Also in human gray matter, the proportion of plasmalogens among total ethanolamine phospholipids is relatively high with about 55 mol%–60 mol% (Han et al., 2001). With regard to the head group, it is of importance that in the entire brain PE[P] is particularly abundant representing 58% of total ethanolamine phospholipids (20% of all phospholipids), whereas PC[P] accounts for only about 1% of total choline phospholipids (0.8% of total phospholipids) (Panganamala et al., 1971; Heymans et al., 1983). In the heart, a similar proportion of ethanolamine phospholipids is present in the plasmalogen form (about 53% of total ethanolamine phospholipids) but, contrasting the brain, 26% of all choline phospholipids are PC[P] (Panganamala et al., 1971; Heymans et al., 1983). Skeletal muscle is comparable to heart tissue in this respect with 48% PE[P] of total ethanolamine phospholipids and 19% PC[P] of total choline phospholipids. On the other hand, the distribution in the kidney more closely resembles that of brain tissue (46% of total ethanolamine phospholipids as PE[P] and 5% of total choline phospholipids as PC[P]). The lowest amounts of plasmalogens are found in the liver with PE[P] representing only 8% of total ethanolamine phospholipids and PC[P] making up 3% of total choline phospholipids (Panganamala et al., 1971; Heymans et al., 1983). Modern lipidome analysis mostly confirms these historical analyses and constitutes an ideal, reliable and valuable technical resource for the detection of changes when comparing different experimental conditions or genotypes. However, due to the differences in the analytical procedure between different lipid classes it can be problematic to compare between individual lipid classes. On the other hand, the use of state-of-the-art lipidomic techniques elucidated an increasing number of less abundant plasmalogen species. From that, it became evident that next to the most common alcohols used by the alkylglyceronephosphate synthase (AGPS)/glyceronephosphate *O*-acyltransferase (GNPAT) complex to form the ether bond, i.e., C16:0, C18:0 and C18:1, also C20:0 is relatively common and many others with chain lengths from 16 to 24 carbon atoms, either saturated or with one or two double bonds, can exist (Amunugama et al., 2021; Azad et al., 2021). Furthermore, recent studies have shown that, apart from the predominant head groups ethanolamine and choline, also serine and inositol can serve as head group (Acar et al., 2007), whereas the broad range of fatty acyl residues at *sn*-2 has long been known.

### 2.2 Plasmalogen levels in different age groups

Plasmalogen levels are not constant in humans but increase and decrease with age. As deficiencies in plasmalogens are associated with a broad spectrum of diseases (see below), it is particularly important to understand which levels are physiological at what age. During gestation, plasmalogen concentration in the cerebrum starts to rapidly increase around the 32nd week together with other important lipids e.g., gangliosides. This increase continues also postnatally until about 6 month of age. Levels in cerebella were described to be generally higher than in the forebrain and continued to rise until the age of 2 years in children (Martínez and Ballabriga, 1978). Another study measured plasmalogen levels in red blood cells of full term neonates showing that they were low and in the range of a peroxisomal biogenesis defect patient. In this study levels doubled in their total value up to the age of 1–5 years (Labadaridis et al., 2009). This strong increase in early childhood was also found by other authors (Rouser and Yamamoto, 1968). In the human central nervous system (CNS), the levels of plasmalogens are relatively low at birth, but rise strongly during early development (Altrock and Debuch, 1968), thus paralleling myelination (Balakrishnan et al., 1961). They were shown to reach a maximum during adulthood, even though the diverse studies come to different conclusions as to the exact age [30 years in (Rouser and Yamamoto, 1968) and 70 years in (Weisser et al., 1997)]. In centenarians, though, lower levels than those measured in a 6-month old embryo were found (Weisser et al., 1997). Another study showed that plasmalogen levels in serum in elderly (65.5 ± 12.0 years of age) were clearly reduced when compared to a younger control cohort (23.5 ± 3.6 years of age) (Maeba et al., 2007). However, in a study comprising 152 elderly people (75.68 ± 0.43 years), the plasma PC[P] levels significantly increased during 90 months of aging and this increase was comparable to that of lyso-PC and lyso-platelet-activating factor (lyso-PAF) (Dorninger et al., 2018).

### 2.3 Plasmalogen levels in disease

The steady state of plasmalogen levels has been described to be altered in many different disorders (Farooqui and Horrocks, 2001; Dorninger et al., 2017). As the initial steps of plasmalogen synthesis take place within peroxisomes, peroxisome biogenesis disorders (PBD; Mendelian Inheritance in Man (MIM) database #601539) including the Zellweger syndrome spectrum (Zellweger syndrome, neonatal adrenoleukodystrophy and infantile Refsum disease), which are caused by either the total absence of peroxisomes or the presence of only peroxisomal ghosts without any luminal enzymes due to impaired protein import, have severely reduced plasmalogen levels. Newborns with PBD are often hypotonic and feed poorly. Further disease characteristics include distinctive facies, congenital malformations, neuronal migration defects associated with neonatal-onset seizures, renal cysts, skeletal abnormalities (chondrodysplasia punctata, rhizomelia) and liver disease that can be severe. Infants with severe PBD are significantly impaired and typically die during the first year of life, usually without developmental progress (Steinberg et al., 2006). The Zellweger syndrome spectrum represents a clinical continuum due to residual import capacity of the affected proteins associated with less severe phenotypes. In Zellweger syndrome spectrum, plasmalogen deficiency contributes to the pathology but as other peroxisomal functions such as peroxisomal ß-oxidation and peroxisomal ɑ-oxidation etc. are disturbed as well, it is difficult to attribute individual pathologies to a specific impaired biochemical pathway. In contrast, the clinical symptoms can solely be attributed to ether lipid deficiency in patients with single enzyme deficiencies affecting one of the genes related to ether lipid biosynthesis, namely the *GNPAT* and the *AGPS* genes (for details see chapter 3.1.1.1 *Peroxisomal steps*), which cause RCDP type 2 (MIM #222765) and RCDP type 3 (MIM #600121), respectively, both of which are inherited autosomal recessively. In patients with the most severe classical form of RCDP, rhizomelia and chondrodysplasia punctata can be detected as early as 18 weeks of gestation by routine ultrasound (Krakow et al., 2003). Patients present with profound rhizomelia, growth retardation, facial dysmorphia, congenital cataract, cardiac defects, seizures, developmental delay and contractures (Braverman and Moser, 2012). As in the Zellweger syndrome spectrum, also the clinical spectrum of RCDP can vary depending on the extent of residual ether lipid synthesis, which can then lead to atypical forms of RCDP without rhizomelia and with only mild growth retardation, subtle facial dysmorphia, congenital cataract and developmental delay (Fallatah et al., 2021). Mutations in the peroxisomal matrix protein import receptors peroxin (PEX) 7 and PEX5L, both required for the import of AGPS into the peroxisome, are the genetic causes for RCDP type 1 (MIM #215100) and RCDP type 5 (MIM #616716), respectively. In the severe form, RCDP types 1, 2, 3, and 5 are clinically indistinguishable. Inherited mutations in the *FAR1* gene causing reduction or complete loss of FAR1 activity result in peroxisomal FAR1 deficiency (MIM #616154, also referred to as RCDP type 4), which can be clinically distinguished from the other forms of RCDP by the absence of chondrodysplasia punctata or rhizomelia (Buchert et al., 2014; Alshenaifi et al., 2019).

While the molecular basis for the plasmalogen reduction is obvious for all these peroxisomal disorders, the observed reduction in non-peroxisomal disorders like Alzheimer’s disease is still under investigation. Presumably, several components contribute to the observed changes in the plasmalogen levels. The causes may also differ depending on which disease and which tissue is investigated. In Alzheimer’s disease, the most common neurodegenerative disease and cause of dementia worldwide, considerably reduced levels of PE[P] (Guan et al., 1999; Han et al., 2001; Kou et al., 2011; Wood et al., 2015) and PC[P] (Grimm et al., 2011a; Igarashi et al., 2011) have been observed in *post mortem* brain tissues. The findings are consistent and were observed in several brain areas as well as in gray and white matter tissues. Interestingly, Kou et al. could demonstrate that in the very same gray matter regions, where plasmalogens were decreased, also other peroxisomal parameters such as peroxisomal density in neurons were affected and an increased concentration of very long-chain fatty acids, which are normally degraded in peroxisomes, has been observed (Kou et al., 2011). Thus, it might well be that different parameters such as synaptic loss (changing lipid composition) as well as impaired peroxisomal functions are causative for the observed reduction in plasmalogens in tissues of patients with Alzheimer’s disease. The amount of plasmalogen reduction in *post mortem* gray matter tissue correlates with the severity of the disease measured as cognitive decline (Han, 2005) or neuropathological staging (Kou et al., 2011). Also in two different mouse models of Alzheimer’s disease, reduced levels of plasmalogens have been described (Fabelo et al., 2012; Tajima et al., 2013). Whereas in the brain tissues of Alzheimer’s disease patients the majority of plasmalogen species are reduced, in blood of Alzheimer’s disease patients only selected plasmalogen species are reduced (Goodenowe et al., 2007; Yamashita et al., 2016; Huynh et al., 2020). Moreover, some of these plasmalogen species, whose levels were observed to be altered, have been suggested as biomarkers for cognitive decline (Mapstone et al., 2014). Interestingly, a recent study also detected reductions of individual plasmanylcholine (PC[O]) and PE[O] species next to PC[P] and PE[P] (Huynh et al., 2020). Another longitudinal population-based birth cohort study demonstrated alterations in the plasma levels of specific choline phospholipids in Alzheimer’s disease that mimic accelerated aging (Dorninger et al., 2018). Also in the plasma of autistic patients the levels of total plasmalogens were found to be reduced by about 15%–20% in two independent studies (Bell et al., 2004; Wiest et al., 2009) and PE[P] were decreased by about 15% in the brain of a rat model of autism (Thomas et al., 2010). In the case of autism spectrum disorders it is interesting to notice that mutations in the *PEX7* gene have been identified using whole exome sequencing (Yu et al., 2013) in agreement with the finding that RCDP patients with a milder (nonclassical) form often present with hyperactivity (Fallatah et al., 2021). Interestingly, also the mouse models for RCDP types 1 and 3 show hyperactive behavior as measured in the open field test (Dorninger et al., 2019b; Fallatah et al., 2020). Moreover, a detailed characterization of the *Gnpat*-deficient mouse model for RCDP type 3 revealed a complex behavioral phenotype mimicking several aspects of human psychiatric disorders (Dorninger et al., 2019a). In this context, it is particularly remarkable that there is a direct correlation between plasmalogen levels and hyperactivity, as demonstrated in a mouse model series with varying degrees of Pex7 deficiency (Fallatah et al., 2022). Thus, there is growing evidence that reduced plasmalogen levels might be associated with hyperactivity in autism spectrum disorders but possibly also in Alzheimer’s disease where hyperactivity is also a commonly observed feature.

Also in other disorders like Parkinson’s disease (Hershkowitz and Adunsky, 1996; Dragonas et al., 2009; Fabelo et al., 2011) and Down syndrome (Murphy et al., 2000; Bueno et al., 2015), reduced levels of plasmalogens in plasma and gray matter have been described. Similarly, plasmalogen levels have been reported to be altered in schizophrenia. However, elevated levels of some PC[P] and PE[P] species have been described in the frontal cortex of patients (Wood et al., 2014), whereas reduced plasmalogen levels were detected in plasma (Kaddurah-Daouk et al., 2012).

## 3 Regulation of plasmalogens on the cellular level

### 3.1 Plasmalogen biosynthesis

#### 3.1.1 Biosynthesis of plasmalogens

##### 3.1.1.1 Peroxisomal steps

**FIGURE 1:**
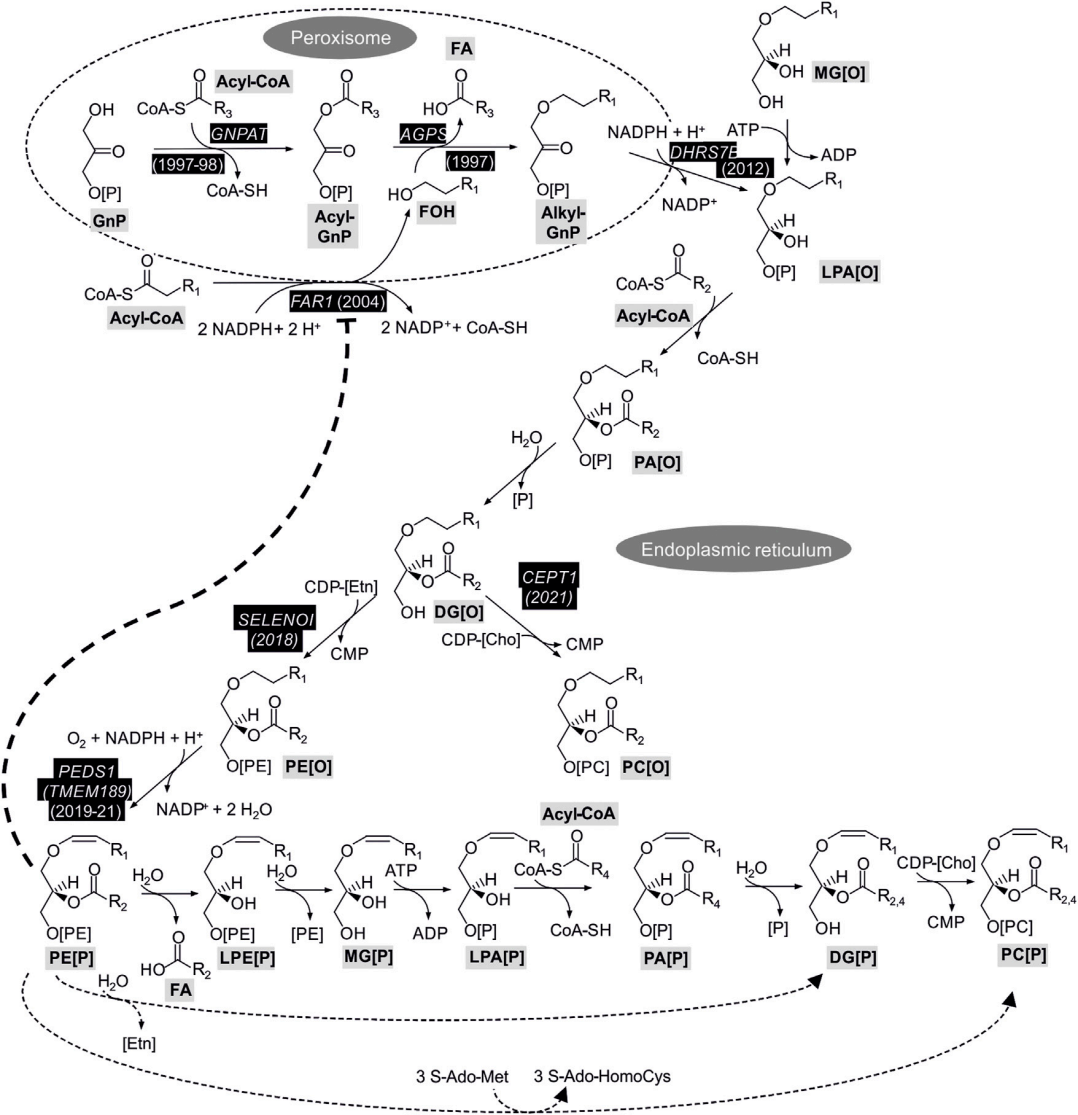
Enzymatic reactions and coding genes with confirmed role for plasmalogen biosynthesis. Gene symbols shown in white on black background indicate genes with experimental evidence for a role in plasmalogen biosynthesis with the year of publication of cloning or sequence assignment. For the steps without a gene assignment it is not clear to what extent genes catalyzing the reactions for ester (diacyl) lipids are also important for ether lipids. The bold dotted line indicates the feedback inhibition of plasmalogens on FAR1 protein stability which is detailed in a dedicated paragraph in chapter 3.1.2 *Regulation of plasmalogen biosynthesis*. The dotted lines in the bottom of the Figure represent routes of biosynthesis alternative to the main route connected by continuous lines. 1-O-Alkenyl-2-acyl-sn-glycerol (DG[P]) and PC[P] carry different side chains at sn-2 (R2 or R4) depending on the route taken from PE[P] (see text for details). The abbreviations of the metabolites follow the guidelines suggested for shorthand notation of mass spectrometry-derived lipid structures (Liebisch et al., 2020). Cho, choline; Etn, ethanolamine; FA, fatty acid; FOH, fatty alcohol; S-AdoMet, S-adenosyl-methionine; S-Ado-HomoCys, S-adenosyl-homocysteine.

Enzymatic reactions of plasmalogen biosynthesis and their coding genes together with the year of gene assignment are shown in Figure 1. Biosynthesis of plasmalogens starts in peroxisomes and is completed at the endoplasmic reticulum (ER) (Nagan and Zoeller, 2001). In a first peroxisomal step, the glycolysis metabolite glycerone phosphate (GnP, previously called dihydroxyacetone phosphate) is acylated by GNPAT (EC 2.3.1.42; encoded by the *GNPAT* gene; (Thai et al., 1997; Ofman et al., 1998)) to yield acylglycerone phosphate (acyl-GnP). Subsequently, the acyl group is exchanged to an alkyl group by AGPS (E.C. 2.5.1.26; encoded by the *AGPS* gene; (de Vet et al., 1997)) by incorporation of a fatty alcohol to yield alkylglycerone phosphate (alkyl-GnP). The fatty alcohol is supplied by alcohol-forming FAR [E.C. 1.2.1.84; encoded by *FAR1* and *FAR2*; (Cheng and Russell, 2004)]. Due to its tissue distribution and enzymatic parameters, the *FAR1* gene product is considered to be more important for ether phospholipid biosynthesis (Cheng and Russell, 2004). Alkylglycerone phosphate is subsequently reduced to 1-*O*-alkyl-*sn*-glycero-3-phosphate (alkyl-lysophosphatidic acid, LPA[O]), which is the ether analog of lysophosphatidic acid (LPA), by the action of acylglycerone phosphate reductase [E.C. 1.1.1.101, previously called acyl/alkyl dihydroxyacetone phosphate reductase; encoded by *DHRS7B*; (Lodhi et al., 2012)]. The DHRS7B protein is associated with the peroxisomal membrane and the ER (Honsho et al., 2020) and the product is released to the ER.

##### 3.1.1.2 Steps at the ER

The peroxisomal steps of plasmalogen biosynthesis can be bypassed by feeding of cells with (mono)alkylglycerol (MG[O]), which is phosphorylated to LPA[O]. At the ER the pathway proceeds downstream of LPA[O] similarly to the classical 1-*O*-acyl glycerophospholipids by acylation at *sn-*2 to alkyl phosphatidic acid (PA[O]) followed by cleavage of the phosphate from *sn-*3 to 1-*O*-alkyl-2-acyl-*sn*-glycerol (DG[O]). It is assumed that the respective enzymes for diacyl glycerophospholipids also act on the analogous ether lipids. DG[O] is then converted to PE[O] by ethanolamine phosphotransferase (E.C. 2.7.8.1) using CDP-ethanolamine (CDP-[E]). In contrast to the previous two steps, in this case the gene important for this step of plasmalogen synthesis is known, i.e., *SELENOI.* The underlying evidence comes from studying a rare inherited disease where this gene was affected, leading to severe complicated hereditary spastic paraplegia, sensorineural deafness, blindness and seizures (Horibata et al., 2018). Interestingly, the levels of PE[P] were more dramatically reduced in the patients’ fibroblasts than that of the classical ester form PE. Due to the block in *SELENOI*, PC[O] (see Figure 1) was increased in the cells. The gene product of *SELENOI*, ethanolamine phosphotransferase 1, is not the only protein known to catalyze this reaction, it can also be supported by the product of *CEPT1* (choline/ethanolamine phosphotransferase 1)*,* but patient data clearly show the prominent role of *SELENOI* for the biosynthesis of plasmalogens (Horibata et al., 2018). Knockout of *SELENOI* in human embryonic kidney (HEK293) cells confirmed its essential role for plasmalogen biosynthesis (Horibata et al., 2020). SELENOI showed a preference for synthesis of PE[O] with longer polyunsaturated side chains at *sn*-2 (Horibata et al., 2020). This may contribute to the preferential occurrence of these long polyunsaturated side chains at *sn*-2 in plasmalogens (Nagan and Zoeller, 2001). The enzyme encoded by *CEPT1*, on the other hand, has been shown to be the major enzyme responsible for the formation of PC[O] in HEK293 cells (Horibata and Sugimoto, 2021). The *CEPT1* gene product prefers shorter more saturated side chains (Horibata et al., 2020), which are also found at *sn*-2 of PC[O] in mouse tissues (Oemer et al., 2020). *CHPT1* is another gene encoding a choline phosphotransferase enzyme capable of attaching choline to DG[O] to form PC[O] like CEPT1. Although of minor importance in quantitative terms in HEK293 cells, the *CHPT1* gene product is remarkable in displaying a preference for the formation of PC[O] with longer unsaturated side chains (Horibata and Sugimoto 2021). Subsequent to *SELENOI*, PEDS1 (E.C. 1.14.19.77, former gene symbol: *TMEM189*) introduces the crucial vinyl ether bond to yield the first plasmalogen in the pathway, i.e., PE[P]. The *PEDS1* gene was recently assigned independently by three groups, one studying a bacterial light response (Gallego-García et al., 2019), one by a dedicated strategy to assign the gene (Werner et al., 2020), and one with a systematic strategy to assign functions to genes with unknown function by looking for co-essentiality of genes in a dataset of 485 human cell lines (Wainberg et al., 2021). PC[P] cannot be formed directly from PC[O] by PEDS1-mediated desaturation, but is formed from PE[P] via several steps yielding the exchange of the phosphoethanolamine at *sn-*3 to phosphocholine (main route, connected by continuous lines in Figure 1) (Lee, 1998). Two further possibilities for the conversion of PE[P] to PC[P], which may also differ between tissues and physiological states of the cells (Nagan and Zoeller, 2001), are illustrated by dotted lines in Figure 1. The routes differ in the retention or exchange of the side chain at *sn-*2 (R_2_ versus R_4_). In the route indicated by a dotted line at the very bottom of the Figure, PE[P] is converted to PC[P] by the action of an N-methyltransferase (Mozzi et al., 1989). PE N-methyltransferase, however, has been found to occur mainly in the liver (Vance et al., 1997), an organ with very low plasmalogen content. Using radiolabeled precursors and HL-60 promyelocytic human cells (Blank et al., 1993) and Madin-Darby canine kidney (MDCK) cells (Strum and Daniel, 1993), it was suggested that a mixture of routes occurs in cultured cells, with only a minor contribution of the N-methylation pathway in neonatal rat myocytes (Lee et al., 1991). Alternatively to the routes shown in Figure 1, phospholipase (PL) D cleavage of alkenyl lysophosphatidylethanolamine (LPE[P]) to alkenyl lysophosphatidic acid (LPA[P], without the MG[P] intermediate) or direct head group alcohol exchange from PE[P] to PC[P] (without the DG[P] intermediate) have been suggested (Blank et al., 1993).

##### 3.1.1.3 Specificity of enzymatic reactions for ether versus ester glycerolipid biosynthesis

With respect to the regulation of plasmalogen biosynthesis it is helpful to keep in mind that many enzymes along the pathway also have roles in the metabolism of diacyl glycerophospholipids. Acylation of glyceronephosphate by GNPAT followed by reduction of acylglyceronephosphate by acylglyceronephosphate reductase (the protein product of the *DHRS7B* gene, which can reduce both acylglycerone phosphate and alkylglycerone phosphate) has been shown by labeling experiments to be of major importance for triacylglycerol biosynthesis in the differentiation of 3T3-L1 adipocytes (Hajra et al., 2000). When calcineurin B homologous protein (CHP1) was inactivated in cells, this resulted in inactivation of glycerol-3-phosphate acyltransferase 4 (GPAT4), yielding a dependence of ester glycerolipid biosynthesis on GNPAT (Zhu et al., 2019). On the other hand, reconstituting a Chinese hamster ovary cell line with GNPAT deficiency by overexpressing GNPAT did not cause gross alterations in non-ether glycerophospholipid concentrations, showing that GNPAT does not normally contribute to non-ether glycerolipid biosynthesis (Liu et al., 2005). Analysis of lipids in cells with a specific deficiency in acylglyceronephosphate reductase suggested a role for this enzyme for ester in addition to ether glycerophospholipids (James et al., 1997). Overexpressing full length or N-terminally truncated acylglyceronephosphate reductase in this cell line altered the subcellular localization of the recombinant enzyme and the preference for restoration of ester versus ether lipids (Honsho et al., 2020). A cell line deficient in AGPS, in contrast, showed no impairment of ester glycerophospholipid biosynthesis (Nagan et al., 1997).

#### 3.1.2 Regulation of plasmalogen biosynthesis

Regulation of plasmalogen biosynthesis has recently been reviewed by Honsho and Fujiki (Honsho and Fujiki, 2017), who have contributed seminal work to this topic. The key regulatory step of plasmalogen biosynthesis is FAR, which is encoded by two genes in man and mouse, i.e., *FAR1* and *FAR2* (Cheng and Russell, 2004). Of these, *FAR1* is of prime importance for plasmalogen synthesis, as it is more widespread in tissue distribution, has substrate specificities resembling the composition of plasmalogens at the *sn*-1 position, and leads to a severe inherited disease in humans with drastically lowered plasmalogen levels when inactivated (Buchert et al., 2014). *FAR2* has a more restricted expression pattern, other substrate specificities, i.e., a preference for saturated fatty acids, and has been suggested to be important for wax ester biosynthesis (Cheng and Russell, 2004). In work with cultured cells, Honsho and others could show that a sensing mechanism in the inner leaflet of the plasma membrane transmits a signal resulting in proteasomal degradation of FAR1 protein when plasmalogen levels are high (Honsho et al., 2010, 2017). This degradation of FAR1 protein can be triggered by PE[P], but not by PE. To which extent precursors or metabolic products of PE[P] can also trigger this regulatory mechanism, remains to be elucidated. A deficiency in the GNPAT or AGPS activities suppressed the formation of plasmalogens and led to an accumulation of fatty alcohols produced by FAR1 both in cultured cells (Honsho et al., 2010) and in plasma and cells from patients with inherited deficiencies in one of these steps (Rizzo et al., 1993). Recently, Ferdinandusse and others described an autosomal dominant disorder caused by *de novo* variants in *FAR1* resulting in uncontrolled ether lipid biosynthesis (Ferdinandusse et al., 2021). Apparently, the mutation inactivates the mechanism of FAR1 protein degradation in the presence of sufficient plasmalogens, leading to a fourfold increase of plasmalogen synthesis in patients’ fibroblasts. Interestingly, the symptoms resemble in some aspects those of plasmalogen deficiency. For example, juvenile cataracts occur in both defective and uncontrolled synthesis of ether lipids. The regulation of plasmalogen biosynthesis by the described sensing of plasmalogens may also limit attempts to increase plasmalogen levels by administration of plasmalogens or precursors. To correct decreased plasmalogens, in murine models of cardiac pathologies (Tham et al., 2018), batyl alcohol (1-*O*-octadecyl-*sn*-glycerol; MG[O] 18:0) was administered (Tham et al., 2018). This resulted in increased amounts of plasmalogens with a 18:0 side chain at *sn*-1, but also in a concomitant reduction of plasmalogens with 16:0 and 18:1 side chains at *sn*-1. Obviously, endogenous biosynthesis of plasmalogens had been downregulated as a result of the treatment, which also failed to correct the cardiac pathologies (Tham et al., 2018).

### 3.2 Plasmalogen remodeling

Plasmalogen remodeling can be discussed from two perspectives, i.e., side chain remodeling on the one hand and remodeling of the polar head group on the other hand.

#### 3.2.1 Side chain remodeling

Side chain remodeling in plasmalogens is a fast process limited to the *sn*-2 position, as cleavage of the vinyl ether-bonded alkyl residue at the *sn*-1 position is an irreversible reaction. Remodeling at the *sn*-2 position consists of rapid deacylation-reacylation steps better known as the Land’s cycle, which also occurs at both *sn*-1 and *sn*-2 in the ester analogues (Shindou et al., 2013; Wang and Tontonoz, 2019).

Deacylation at the *sn*-2 position is the first step in glycerophospholipid remodeling and is accomplished by the PLA_2_ superfamily. This huge enzyme family is divided into six subfamilies, i.e., cytosolic PLA_2_ (cPLA_2_), calcium-independent PLA_2_ (iPLA_2_), secreted PLA_2_ (sPLA_2_), lysosomal PLA_2_, PAF acetylhydrolases, and adipose-specific PLA_2_ (Peng et al., 2021). The first three of these six subfamilies together comprise more than 20 different isoforms (Peng et al., 2021). The PLA_2_ reaction results in a free fatty acid and a lysophospholipid. Besides being important for the remodeling itself, PLA_2_-mediated release of arachidonic acid directly impacts inflammatory processes because it provides the precursor for many bioactive inflammatory mediators like eicosanoids. Arachidonic acid was shown to be specifically introduced into plasmalogens via the remodeling pathway and not by the *de novo* biosynthesis pathway (Ford and Gross, 1994; Yamashita et al., 1997).

Until now, involvement of the type of *sn*-1 chain linkage in determining PLA_2_ selectivity was only scarcely investigated. Functioning of PAF, a pleiotropic factor in many cellular processes including inflammation and the best-described member of the PC[O] class (Demopoulos et al., 1979), is regulated by hydrolysis of the acetyl residue at *sn*-2 leading to its deactivation. It was shown that this specific deacetylation step is accomplished by PAF acetylhydrolase (also known as lipoprotein-associated PLA_2_) (Tjoelker et al., 1995; Mouchlis et al., 2022). Also, 1-*O*-alkyl and 1-*O*-alkenyl ether phospholipids have been proposed to be remodeled by a CoA-independent transacylase, which specifically transfers polyunsaturated C20- and C22-fatty acids (Fleming and Hajra, 1977; Sugiura et al., 1987) explaining the finding that ether-linked phospholipid species specifically accumulate PUFA at their *sn*-2 position. The distribution pattern of this enzyme system, which is mainly located to microsomes of most tissues except liver, mirrors the distribution pattern of ether-linked phospholipids making it a likely candidate for ether phospholipid remodeling (Yamashita et al., 2014). However, no gene has been assigned so far to this proposed CoA-independent transacylase activity. Two additional studies also looked into the influence of the *sn*-1 bond type on substrate specificity of single PLA_2_ enzymes. In 1992, Diez and others investigated a 14 kDa recombinant human synovial fluid PLA_2_ and found a slight preference for acyl over alkyl residues at *sn*-1, whereas a 85 kDa cytosolic PLA_2_ from monocytic cells (also named high molecular weight PLA_2_) did not display a preference for a C16 ester- versus ether-bonded side chain at *sn*-1 (Diez et al., 1992). One year later, Hanel et al. also found that mammalian high molecular weight PLA_2_ accepted 1-stearoyl-2-arachidonyl-*sn*-glycero-3-phosphocholine and 1-*O*-hexadec-1′-enyl-2-arachidonyl-*sn*-glycero-3-phosphocholine equally well as substrate (Hanel et al., 1993). Two calcium-independent PLA_2_ with a preference for plasmalogens were described in the 1990s. The first one purified from canine myocardial tissue was shown to specifically hydrolyze PC[P] and arachidonylated glycerophospholipids (Hazen et al., 1990). The second one purified from bovine brain has a molecular mass of 39 kDa and was shown to preferentially cleave PE[P] (Hirashima et al., 1992). Two decades later, the same group also described a protocol for partial purification of a plasmalogen-selective PLA_2_ from pig brain (Farooqui, 2010). Finally, very recently, a significant step forward was made in our understanding of how the *sn*-1 linkage impacts on PLA_2_ selectivity. Hayashi and co-workers systematically assessed this issue by studying three different PLA_2_ isoforms. They were able to show that for 1-*O*-alkyl ether phospholipids both group IVa cPLA_2_ and group VIa iPLA_2_ had about half the activity as for ester phospholipids (for group IVa cPLA_2_ activity depended strongly on the *sn*-2 fatty acyl side chain, whereas group VIa iPLA_2_ was more permissive in this regard). When testing 1-*O*-alkenyl ether phospholipids, however, preference of group IVa cPLA_2_ was clearly on the side of the plasmalogens and not the ester-bonded lipids with a selectivity for PUFA at the *sn*-2 position, while group VIa iPLA_2_ was equally active towards ester and plasmalogen phospholipids. In contrast to these two PLA_2_ subtypes, the secreted isoform group V sPLA_2_ clearly favors ester lipids over both alkyl and alkenyl ether phospholipids (Hayashi et al., 2022). These results indicate that the enzymes can distinguish between the various *sn*-1 bond types present in glycerophospholipids. Molecular modeling studies also revealed that the carbonyl oxygen in ester lipids interacts with a tryptophan in group V sPLA_2_ allowing for precise accomodation of the ester glycerophospholipids in the catalytic pocket and efficient hydrolysis of the acyl side chain (Hayashi et al., 2022).

The reacylation of ester and ether lysophospholipids is catalyzed by at least 14 different acyltransferases, which are known as acylglycerophosphate acyltransferases (AGPAT), membrane-bound *O*-acyltransferases (MBOAT), lysophosphocholine(ethanolamine) acyltransferases (LPC(E)AT) or more generally as lysophospholipid acyltransferases (LPLAT). As existing names were quite confusing, in part with multiple names for a single protein, a new nomenclature was recently proposed focusing on LPLAT only (Valentine et al., 2022). In the following paragraph we will focus on those isoforms known to impact on ether lipid, including plasmalogen, remodeling by using the updated nomenclature and providing former enzyme names in brackets.

Recently, it was found that LPLAT12 (LPCAT3)-deficient mice presented with disrupted plasmalogen homeostasis. A strong decrease was found in arachidonic (C20:4) and eicosapentaenoic (C20:5) acid *sn*-2 substitutions, which were compensated by other fatty acids (e.g., C22:4) leading to an overall balanced total amount of plasmalogens (Thomas et al., 2018). It is however not clear so far whether LPLAT12 (LPCAT3) contributes to the previously described CoA-independent transacylase system. In PAF remodeling, the acylation step consists of acetylation of lyso-PAF accomplished by LPLAT9 (LPCAT2), but also LPLAT8 (LPCAT1) (Shindou et al., 2007; Harayama et al., 2008). In a recent CRISPR-Cas9-based study investigating the role of ether lipids in the promotion of ferroptotic cell death, LPLAT3 (AGPAT3) emerged as a significant hit. This provided solid evidence that this isoform, already known to incorporate arachidonic and docosahexaenoic acid into ester lysophosphatidic acids (Yuki et al., 2009), is also crucial for generating *sn*-2-PUFA-containing ether phospholipids, which are essential for the susceptibility to ferroptosis (Zou et al., 2020). In previous work, where LPLAT3 (AGPAT3) had also popped up as potential hit in a CRISPR screen, LPLAT12 (LPCAT3) was shown to fuel ferroptosis (Dixon et al., 2015) and this effect was attributed to the enzyme’s preference for arachidonic acid insertion into membrane phospholipids (Shindou and Shimizu, 2009).

#### 3.2.2 Head group remodeling

Besides fast remodeling at the *sn*-2 position, plasmalogens also undergo polar head group remodeling. This exchange was shown to be 300 times faster than *de novo* plasmalogen synthesis - as measured by *sn*-1 alkyl chain incorporation - in studies using tritium-labeled choline or ethanolamine in contracting myocardium. Of note, this is in contrast to diacyl phospholipids, where *de novo* synthesis and remodeling happen at similar rates (Ford and Gross, 1994). Remodeling of PE[P] to PC[P] was proposed to occur through different mechanisms (Figure 1). Experimental evidence provided by Blank and coworkers in 1993 showed, that next to a multistep enzymatic cascade involving a PLA_2_, followed by lysophospholipase D, acyltransferase, phosphohydrolase and cholinephosphotransferase steps [which resembles the *de novo* biosynthetic pathway presented in a comprehensive review (Nagan and Zoeller, 2001) and shown in Figure 1] also a more direct way exists. This involves either base exchange from ethanolamine to choline or a PLC-catalyzed removal of the phosphoethanolamine headgroup and introduction of phosphocholine through a choline phosphotransferase active in HL-60 cells (Blank et al., 1993). The existence of a PLC subtype which accepts plasmalogens as substrates was already proven 8 years earlier (Wolf and Gross, 1985).

Plasmalogens have been attributed a role in cardiolipin remodeling as they are substrates for the main enzyme responsible for producing mature cardiolipins, a transacylase named taffazin (Kimura et al., 2018). In tafazzin-deficient mouse heart, PC[P], arguably the most abundant phospholipid in this tissue, was strongly reduced, while PE[P]was unchanged and FAR1 protein expression was upregulated. This upregulation succeeded in maintaining PE[P] levels but was not able to counteract increased PC[P] degradation (Kimura et al., 2018). Follow-up studies from the same group then showed that other tissues including human brain and lymphoblasts obtained from Barth syndrome patients, characterized by taffazin deficiency, showed a marked reduction in PE[P] but at least in lymphoblasts no upregulation in FAR1 was found (Kimura et al., 2019). Supplementation of taffazin-deficient lymphoblasts with 1-*O*-hexadecyl-*sn*-glycerol, a precursor of plasmanyl and plasmenyl lipids, led to restoration of PE[P] and also cardiolipin levels (Bozelli et al., 2020). As tafazzin does not display a clear preference for certain acyl side chains and the molecular composition of cardiolipins differs from tissue to tissue, a systematic analysis of the impact of tissue phospholipid side chain composition on the cardiolipin side chain composition was performed. By applying machine learning, Oemer and others could show that the presence of high linoleic acid levels in the phospholipid pool was strongly linked to displacement of other fatty acids from the cardiolipin pool. Also oleic acid was a strong driver, however, oleic and linoleic acid inhibited each other’s incorporation. In the brain, which is characterized by a unique fatty acyl side chain composition with many longer-chained essential fatty acids, the class of ether-linked phosphoethanolamine lipids displayed the highest profile similarities to cardiolipins (Oemer et al., 2020).

### 3.3 Regulation of degradation

#### 3.3.1 Routes of degradation

This chapter only focuses on pathways and enzymatic reactions, which lead to degradation of the vinyl ether bond. Strictly speaking, on the level of individual plasmalogen subspecies, also the removal of fatty acyl chains or plasmalogen head groups by PLs represents a way of degradation. However, for further details on species remodeling at the *sn*-2 and *sn*-3 positions, we kindly refer the reader to chapter 3.2 *Plasmalogen remodeling*.

##### 3.3.1.1 Lysoplasmalogenase

Lysoplasmalogenase is an enzyme that acts, as its name suggests, on lysoplasmalogens, meaning that for its degradation of plasmalogens, the previous activity of a PLA_2_ is required for removing the acyl side chain at the *sn*-2 position. The vinyl ether bond of lysoplasmalogens can then be hydrolyzed by lysoplasmalogenase (alternative name: alkenyl hydrolase) thus liberating a long chain fatty aldehyde and glycerophosphocholine/-ethanolamine. Already as early as 1961, a corresponding activity was detected in microsomes isolated from rat brain (Warner and Lands, 1961). Later, the enzyme was successfully purified and characterized in microsomes from rat liver (Jurkowitz-Alexander et al., 1989) and small intestine (Jurkowitz et al., 1999), two tissues, where its expression is reportedly high (Jurkowitz, 2015). However, it took until 2011 to assign a gene to this enzymatic function, when *TMEM86*B was identified as the gene coding for lysplasmalogenase in mammals (Wu et al., 2011). *TMEM86B* produces a transmembrane protein with six predicted transmembrane domains and was, accordingly, purified from membrane fractions. Interestingly, its activity is inhibited by LPA, which does not serve as a substrate (Wu et al., 2011). Unfortunately, LPA[P], the alkenyl analog of LPA, which has also been shown to be a bioactive compound (Dorninger et al., 2020), was not tested in that study. The enzyme encoded by *TMEM86B* accepts lysoplasmalogens with both headgroups, ethanolamine and choline (Wu et al., 2011), as substrates and, presumably, also acts on free alkenylglycerol (Gunawan and Debuch, 1985), a compound produced from lysoplasmalogen by the action of a PLC. Earlier studies, though, report the occurrence of a lysoplasmalogenase activity specific for individual head groups, i.e., for lysoplasmenylcholine (Warner and Lands, 1961; Arthur et al., 1986), thus feeding speculation that, apart from TMEM86B, additional, not yet identified lysoplasmalogenases exist in mammals (Watschinger and Werner, 2013). An obvious candidate is its paralog TMEM86A, a protein of long unknown function, which shares around 40% of its protein sequence with TMEM86B (Jurkowitz et al., 2015) and for which lysoplasmalogenase activity is predicted by the gene ontology (GO) project. No biologic function has been ascribed to the protein encoded by *TMEM86A* until recently. However, just during the production of the present review, a notable study was published demonstrating lysoplasmalogenase activity of TMEM86A in adipocytes and linking it to the development of obesity (Cho et al., 2022). Interestingly, the *TMEM86A* gene was also named in a recent study as a potential regulator of human epidermal keratinocyte differentiation (Zhang et al., 2021). Yet, the molecular mechanism underlying this finding remains unclear.

Interestingly, a protein with lysoplasmalogenase activity related to TMEM86B was discovered in bacteria of the *Legionella* genus. This is particularly remarkable considering that these bacteria do not possess plasmalogens themselves. However, it was speculated that the lysoplasmalogenase represents some kind of defense mechanism against host lysoplasmalogens, which could induce bacterial lysis (Jurkowitz et al., 2015). Just very recently, a similar mechanism was also revealed for another bacterial pathogen, *Mycobacterium tuberculosis* (Jurkowitz et al., 2022).

##### 3.3.1.2 Plasmalogenase

The term plasmalogenase designates an enzyme with the ability to cleave the vinyl ether bond of plasmalogens. In contrast to the lysoplasmalogenase reaction (cf. chapter 3.3.1.1 *Lysoplasmalogenase*), the *sn*-2 position remains acylated prior to the action of plasmalogenase. A corresponding activity has been described in early studies on plasmalogens in mammalian brain and heart (Ansell and Spanner, 1968; Yavin and Gatt, 1972; D’Amato et al., 1975; Arthur et al., 1985) but proteins catalyzing the reaction could not be revealed for decades. Finally, in a hallmark study, cytochrome C, a mitochondrial enzyme most famous for its role in the respiratory chain and in apoptosis, was discovered as the first enzyme to exhibit plasmalogenase activity (Jenkins et al., 2018). The authors demonstrated that the peroxidase properties of cytochrome C lead to efficient hydrolytic cleavage of the vinyl ether bond under utilization of molecular oxygen. For activation of the plasmalogenase activity, though, the presence of either negatively charged lipids like cardiolipin and H_2_O_2_ or oxidized cardiolipin (or a similar lipid) are required. Even though some of the older reports indicated enzymatic activity only towards PE[P] (maybe due to their higher abundance) (Arthur et al., 1986), Jenkins et al. confirmed that both PE[P] and PC[P] are readily degraded by myocardial cytochrome C (Jenkins et al., 2018).

Yet, similarly as for lysoplasmalogenase, it remains a matter of debate if, apart from cytochrome C, also other enzymes possess plasmalogenase activity in mammals. One puzzling aspect is the fact that early reports described a considerable fraction of plasmalogenase activity to be located in the microsomal fraction after subcellular fractionation (Ansell and Spanner, 1968; Arthur et al., 1985), which is in apparent contradiction to the subcellular localization ascribed to cytochrome C. However, prominent activity was described in the mitochondrial fraction as well (Ansell and Spanner, 1968). Later, also cytosolic plasmalogenase activity was demonstrated in guinea pigs (McMaster et al., 1992), which would also fit to the idea of cytochrome C as responsible enzyme, as cytochrome C can be released into the cytosol under stress conditions (Garrido et al., 2006). Furthermore, the plasmalogenase activity of cytochrome C requires highly specific conditions, i.e., the presence of O_2_ and H_2_O_2_ or oxidized cardiolipin. Altogether, these facts may promote speculations that further enzymes capable of cleaving plasmalogens have to exist to explain the wide abundance of plasmalogenase activity as detailed in early studies.

##### 3.3.1.3 Other enzymatic pathways

Myeloperoxidase is a lysosomal enzyme that is mainly expressed in neutrophils and monocytes and is an important regulator of inflammatory processes. Upon availability of H_2_O_2_, it converts chloride (Cl^−^) and bromide (Br^−^) ions to HOCl and HOBr, respectively. The vinyl ether bond of plasmalogens is susceptible to oxidation by both these compounds, resulting in the formation of lysolipids and the release of 2-halo fatty aldehydes (Albert et al., 2001), which themselves activate downstream signaling pathways and modulate immune reactions (Ebenezer et al., 2020). In the brain, upregulation of myeloperoxidase in response to microbiota attack has been implicated in neurodegeneration. The fact that HOCl-mediated oxidative attack of plasmalogens has been also proven *in vivo* in the mouse brain fueled speculation that plasmalogen degradation and subsequent defects in synaptic transmission are part of the underlying mechanism (Ullen et al., 2010).

Other than the hydrolases, which are important for the degradation of plasmalogens, plasmanyl lipids, which in contrast to plasmalogens do not harbor a vinyl ether bond next to the ether linkage, are degraded by the mixed function oxidase alkylglycerol monooxygenase (AGMO, E.C. 1.14.16.5) in the presence of the cofactor tetrahydrobiopterin (Watschinger et al., 2010). AGMO modulation in a knockdown macrophage cell line impacted the overall lipidome leading to increases in plasmanyl and plasmenyl lipids as well as strong reductions in glycosylated ceramides and cardiolipins (Watschinger et al., 2015). Recently, we established an AGMO knockout mouse, which presents with no obvious phenotype in unchallenged conditions (Sailer et al., 2021).

The fatty aldehydes resulting from the various degradation pathways, as outlined above, are toxic for cells due to their high reactivity in forming adducts with protein side chains and lipids and are therefore efficiently oxidized to the corresponding fatty acids by fatty aldehyde dehydrogenase (Weustenfeld et al., 2019). The fatty acids are then either catabolized for energy production or used in anabolic processes.

##### 3.3.1.4 Non-enzymatic degradation of plasmalogens

Plasmalogens are susceptible to reactive oxygen species (ROS) that have been proposed to attack the vinyl ether double bond leading to enzyme-independent plasmalogen degradation and production of aldehydes. These aldehydes were either (n-1) carbons shorter than the original fatty alcohol or belonged to the class of α-hydroxyaldehydes. Both these product types are compatible with oxidative breakage of the vinyl ether double bond (Stadelmann-Ingrand et al., 2001). In contrast to diacyl phospholipids, plasmalogens have been discussed to be more prone to oxidation by such oxygen radicals (Khaselev and Murphy, 1999; Broniec et al., 2011). This feature has repeatedly evoked discussions about whether plasmalogens act as cellular antioxidants. For example, data from the Wanders group showed no changes in plasmalogen levels in cultured human skin fibroblasts incubated with or without intracellular ROS generator (Jansen and Wanders, 1997) and also Broniec et al. showed, 6 years after their first investigation (Broniec et al., 2011), that antioxidant properties in plasmalogens are not solely dependent on the vinyl ether (Broniec et al., 2017). In contrast, Zoeller et al. found that plasmalogen-deficient RAW264.7 cells were more susceptible to chemical hypoxia and supplementation with PE[P] reverted this hypersensitivity back to control levels (Zoeller et al., 1999) supporting the hypothesis of plasmalogens being antioxidants. In lupus erythematosus patients, which have elevated oxidative stress levels, it was found that plasmalogens were selectively decreased and *sn*-1 LPE (deacylated at the *sn*-1 position) and 4-hydroxy-2(E)-nonenal were increased (Hu et al., 2016).

#### 3.3.2 Turnover of plasmalogens

A variety of studies have focused on different remodeling steps of plasmalogens (cf. chapter 3.2 *Plasmalogen remodeling*), but information on the turnover, i.e., the rate of degradation of the vinyl ether bond, is scarce. Although turnover rates most likely diverge strongly between organs and tissues, the few available data that have been gathered were derived almost exclusively from the brain. Early reports stated a relatively rapid turnover of brain plasmalogens, even of those in the myelin compartment (Horrocks and Fu, 1978), but indicated that half lives of PE[P] were longer than those of the corresponding diacyl phospholipids, even though exact numbers varied depending on the type of radioactively labeled precursor used (Miller et al., 1977). Later however, more detailed investigations involving intravenous infusion of radioactively labeled hexadecanol into anesthetized rats identified two different pools of brain PE[P] (Rosenberger et al., 2002) thus confirming earlier hypotheses (Freysz et al., 1969; Goracci et al., 1975): One is the metabolically less active myelin pool and the other a more dynamic pool of gray matter plasmalogens, which is turned over much more rapidly (Rosenberger et al., 2002) and may be a player in synaptic fusion and constriction processes like those in neurotransmission (Dorninger et al., 2019b). Strikingly, the half lives calculated in that study are in the magnitude of minutes, whereas another report estimated turnover times for myelin and microsomal PE[P] in the range of 4–58 days (Miller et al., 1977). The reason for this discrepancy may lie in the type or properties of the tracer used (Rosenberger et al., 2002) but illustrates that reliable absolute numbers for plasmalogen turnover are difficult to determine.

Notably, elaborate studies from the 1970s indicated that PC[P], which are comparatively rare in brain tissue, are metabolized more rapidly compared to their ethanolamine counterparts (Goracci et al., 1976). This is in accordance with the idea that the two subclasses fulfill fundamentally different roles in the brain. Whereas a large fraction of the much more abundant PE[P] likely are essential for membrane and myelin structure associated with a lower rate of turnover, PC[P] could serve other roles like acting as signaling mediators (Dorninger et al., 2020) and may thus be more short-lived.

#### 3.3.3 Contributions of the different pathways and their regulation

The general view in literature appears to be that plasmalogen levels and composition are rather regulated via biosynthesis and remodeling than via cleavage of the vinyl ether bond. However, the reason for this may simply lie in the much higher number of studies on the former topics than on plasmalogen degradation. Actually, due to the fact that the genes and enzymes determining plasmalogen degradation have only been identified in recent years, knowledge on the interplay and regulation of these pathways is still in its infancy. Accordingly, the actual contributions of lysoplasmalogenases, plasmalogenases and other mechanisms to the turnover of plasmalogens can only be hypothesized, but is not yet supported by hard facts. A remarkable observation in this context is the seemingly reciprocal relationship between plasmalogen levels and lysoplasmalogenase activity (Gunawan and Debuch, 1981; Jurkowitz-Alexander et al., 1989; Wu et al., 2011). Most strikingly, lysoplasmalogenase expression and activity are high in the liver, an organ with particularly low amounts of plasmalogens, whereas heart and brain are characterized by both high plasmalogen levels and comparatively low lysoplasmalogenase activity. In addition, overexpression of *TMEM86B*, the gene coding for lysoplasmalogenase, in cultured cells resulted in lowered plasmalogen levels (Wu et al., 2011). This led several authors to speculate that lysoplasmalogenase is the major metabolic route for plasmalogen degradation or even for regulation of total plasmalogen levels (Braverman and Moser, 2012; Meikle and Summers, 2017).

In turn, a similar reciprocal association as for lysoplasmalogenase between expression and plasmalogen levels cannot be stated for cytochrome C, the only known plasmalogenase in mammals. *CYCS*, the gene encoding cytochrome C, is abundantly expressed in cardiac tissue, where its plasmalogenase activity was originally identified, but its expression is relatively low in the liver.

On the other hand, in the brain, plasmalogenase activity has been found to parallel the myelination process (Horrocks et al., 1978) and to increase in demyelinating lesions (Ansell and Spanner, 1968; Horrocks et al., 1978), possibly to get rid of phospholipids associated with myelin debris. However, it still needs to be established if cytochrome C acts as plasmalogenase in all tissues and/or if possibly other proteins capable of degrading plasmalogens exist.

What seems clear is that oxidative stress favors cleavage of the vinyl ether bond via different routes. Direct oxidative attack of the vinyl ether bond (see chapter 3.3.1.4 *Non-enzymatic degradation of plasmalogens*), increased activity of myeloperoxidase in certain cell types (see chapter 3.3.1.3 *Other enzymatic pathways*) and generation of oxidized phospholipids and H_2_O_2_ to support the plasmalogenase activity of cytochrome C (see chapter 3.3.1.2 *Plasmalogenase*) all take place under oxidative stress conditions. From this perspective, it is not surprising that a variety of human pathological conditions, which are associated with accumulation of reactive oxygen species, go along with a decrease of plasmalogen levels, like Alzheimer’s disease (Han et al., 2001), spinal cord ischemia or hyperlipidemia (Brosche and Platt, 1998). For example, in the case of Alzheimer’s disease, increased cytochrome C-mediated decay of plasmalogens as a consequence of oxidative stress has been hypothesized to be a driver of pathology (Jenkins et al., 2018). Similar mechanisms might be at work in other diseases, where H_2_O_2_ is excessively produced and serves as foundation for the plasmalogenase activity of cytochrome C. However, in mitochondria, where cytochrome C is normally localized, H_2_O_2_ is continuously produced by the respiratory chain (Sies, 2014) and, thus, probably present in every metabolically active cell. On the other hand, oxidized cardiolipin (another prerequisite for the plasmalogenase reaction mediated by cytochrome C) is apparently a marker of stress (Ji et al., 2012; Vähäheikkilä et al., 2018; Pizzuto and Pelegrin, 2020) and causes a domino effect eventually leading to neuronal death. Accordingly, future studies are needed to elucidate if cytochrome C acts as plasmalogenase to a relevant extent under physiological conditions or is only active upon excessive stress. Only then, its contribution to plasmalogen degradation in healthy cells and tissues can be assessed and put in the context of the other known routes of degradation, particularly the lysoplasmalogenase pathway.

## 4 Regulation of plasmalogen distribution across tissues in mammals

Even though humans (and most animals) have their own machinery to produce plasmalogens, these lipids can also be taken up from external sources. In this chapter, we discuss options for the supplementation with plasmalogens and current knowledge on how orally ingested plasmalogens are metabolized. Furthermore, we address the question, if and how plasmalogens are distributed to different tissues in mammals.

### 4.1 Dietary intake of plasmalogens

#### 4.1.1 Plasmalogen content of conventional food

Plasmalogen ingestion via the diet happens already in early infancy, because several analytical studies have provided proof for the presence of plasmalogens and also lysoplasmalogens in breast milk from various mammalian species, including humans. Remarkably, the studies come to different conclusions as to the levels of the individual subclasses with one report stating that PC[P] account for the majority of human breast milk plasmalogens (Song et al., 2021), whereas other studies find PE[P] as the most abundant subclass (Garcia et al., 2012; Alexandre-Gouabau et al., 2018). Compared with other phospholipid classes (e.g., PE), PE[P] of breast milk has been described as particularly rich in PUFA (Moukarzel et al., 2016), which could be a major contributor to proper development of the newborn, given the importance of these fatty acids for brain formation and function (Bazinet and Layé, 2014). At later developmental stages, plasmalogen is mainly taken up by meat consumption. A comprehensive analysis of various meat subtypes revealed highest plasmalogen amounts in meat from livestock (especially beef, lamb and chicken) and considerably lower levels in different types of seafood, including fish and mussels (Wu et al., 2021). For meat bonvivants among the readers, it has been reported that meat derived from certain large deer species, i.e. moose and caribou, contains particularly high amounts of plasmalogens and lysoplasmalogens (Pham et al., 2021). Manipulation of foodstuff, like boiling, frying, or freeze-thawing, reportedly reduces plasmalogen content (Wu et al., 2020; Chen et al., 2022), which may in part be due to the propensity of these lipids for oxidation (cf. chapter 3.3.1.4 *Non-enzymatic degradation of plasmalogens*).

Not only plasmalogen content but also head group and side chain composition vary between meat from different species. Interestingly, analysis of the plasmalogens derived from the meat of certain marine invertebrates (shellfish) revealed high amounts of species with a serine headgroup, which is uncommon in mammalian tissue (Kraffe et al., 2004; Wang et al., 2021). Furthermore, a recent study investigating physiological effects of plasmalogen treatment detected considerably higher levels of docosahexaenoic acid and eicosapentaenoic acid in plasmalogens isolated from scallops compared with those of chicken, whereas oleic acid was enriched in chicken plasmalogens. Accordingly, scallop-derived plasmalogens showed more beneficial effects on memory tasks, when fed to mice (Hossain et al., 2022).

An essential question in the context of dietary plasmalogens is their metabolization and potential degradation in the course of the digestion process. In particular, the strongly acidic milieu in the stomach poses a considerable problem to the stability of plasmalogens, as the vinyl ether bond has been found to be sensitive to acid treatment already decades ago (Jones and Wood, 1964; Frosolono and Rapport, 1969). However, *in vitro* experiments exposing plasmalogens (embedded in sucrose-casein-based food pellets) for 1 h to conditions mimicking those in the mammalian stomach did not lead to plasmalogen degradation at a relevant rate. Likewise, the vinyl ether structure was largely preserved after incubation of emulsified plasmalogens with the intestinal contents of rats (Nishimukai et al., 2003). The exact extent of plasmalogen preservation in the stomach, though, may well depend on the exact pH of gastric juice. This can be deduced from other, yet unpublished data gathered by NMR spectroscopy, suggesting that plasmalogens can persist for 1 h at pH 2, but the rate of degradation increases considerably under lower pH and with increasing time (http://www.oilsfats.org.nz/wp-content/uploads/2016/02/Dawn-Scott-Nelson-talk-Nov-2016.pdf). Altogether, these data support the hypothesis that plasmalogens are not completely broken down in the alimentary tract after oral ingestion. Instead, at least a part of ingested plasmalogens, presumably after being packaged into chylomicron particles like other phospholipids (Iqbal and Hussain, 2009), is absorbed from the intestine into the lymph, as demonstrated by studies using duodenal infusion in rats (Hara et al., 2003). Remarkably, this process has been shown to be more effective for PC[P] than for PE[P] (Nishimukai et al., 2011). Furthermore, the *sn*-2 position appears to undergo remodeling with a clear preference for enrichment of arachidonic acid during absorption (Takahashi et al., 2020). Following the absorption process, chylomicrons likely serve as vehicles for plasmalogens to enter the circulation via the lymphatic system. In spite of these considerations, compared with endogenous biosynthesis, the contribution of the diet to tissue plasmalogen levels of mammals is supposed to be very low.

#### 4.1.2 Plasmalogen supplementation as therapeutic strategy

Next to the ingestion via conventional dietary sources, the proactive supplementation with plasmalogens or their precursors represents an additional external source of plasmalogens, either as therapeutic strategy against pathological conditions or as a beneficial complement of the normal diet. Originally, the main intention for such an approach was the identification of treatment options for peroxisomal disorders with inborn ether lipid deficiency, as no cure for these dramatic diseases has been found yet. The most straightforward and also affordable strategy in this respect represents the supplementation with alkylglycerols like chimyl alcohol or batyl alcohol. These compounds contain a pre-formed ether bond and thus circumvent the peroxisomal steps of ether lipid biosynthesis, which are impaired in peroxisomal disorders associated with ether lipid deficiency. They are, therefore, readily converted to plasmalogens after oral ingestion and distributed to all peripheral organs, but not (at least not in substantial amounts) the brain, as shown by experiments in humans, mice and rats (Das and Hajra, 1988; Das et al., 1992; Brites et al., 2011; Paul et al., 2021). However, only limited and selective functional improvements have been reported after alkylglycerol treatment in ether lipid-deficient mouse models (Brites et al., 2011; Todt et al., 2020) and no successful treatment with these compounds has yet been reported for human patients. Only recently, an unusual type of alkylglycerol with a C14 chain (tetradecylglycerol) has shown promising results particularly in the rescue of the myelination deficits in ether lipid-deficient animals and in *in vitro* assays (Malheiro et al., 2019). Interestingly, therapeutic application of alkylglycerols was also mentioned in other contexts apart from the treatment of ether lipid deficiency. Specifically, these substances are associated with antioxidative activity, immune system stimulation, anti-tumorigenic agents or facilitation of transport of other therapeutics (e.g., chemotherapeutics) across the blood-brain barrier (Erdlenbruch et al., 2003; Iannitti and Palmieri, 2010; Poleschuk et al., 2020).

In more recent years, with a continuously rising number of reports of pronounced plasmalogen deficits in human patients with common neurological disorders, particularly Alzheimer’s disease, the interest in plasmalogens and their derivatives as a therapeutic option has markedly increased (Paul et al., 2019; Bozelli and Epand, 2021). Several preclinical studies have suggested that application of plasmalogens or their precursors has beneficial effects on cognitive or behavioral performance in mice and rats (Yamashita et al., 2017; Che et al., 2018; Fallatah et al., 2020; Hossain et al., 2022). A small-scale clinical study in Alzheimer’s disease human patients even identified cognitive improvements in a subset of patients after oral administration of scallop-derived plasmalogens (Fujino et al., 2017). All these results are particularly astounding given the fact that none of the treatment strategies has succeeded in producing a measurable increase in brain plasmalogen levels, likely due to the fact that plasmalogens do not or only hardly cross the blood-brain barrier. Nevertheless, plasmalogens may unfold physiological actions relevant for the brain even without actually reaching it. One possibility that was suggested is a modulation of the gut microbiome (Hossain et al., 2022), which could also influence brain functions (Morais et al., 2021). Alternatively the supplemented plasmalogens could simply serve as vehicles for PUFA, which can be cleaved off the *sn*-2 position and reach the brain independently, where they may exert beneficial effects. Also, it is conceivable that minor amounts of plasmalogens or ether lipid species derived from them reach the brain without a measurable change in total levels, but still have biological effects, e.g., in signal transduction (Dorninger et al., 2020). Accordingly, plasmalogens are seen by some as a promising therapeutic target for the treatment of neurological diseases. In some markets, plasmalogens are even advertised as dietary supplements that should be taken prophylactically in order to avoid cognitive decline. However, an experimental proof for such an approach is not yet available.

With increasing commercial interest to exploit the therapeutic potential of plasmalogens, the strategies to increase their levels in humans have become manifold: Several different companies provide plasmalogens themselves, either as an oil formulation or encapsulated. Also, the individual formulations differ in their manufacturing; synthetically produced plasmalogens as well as plasmalogens extracted from natural sources, mostly from marine species like scallops, are available. Some commercially available plasmalogens show a specific chain distribution at *sn*-2 (e.g., DHA- or EPA-enriched), whereas others represent the whole spectrum of subspecies extracted from a certain source. In several marketed products, the actual plasmalogen amounts are surprisingly low and likely do not even exceed the levels present in meat products that are a normal part of the diet. Other providers pursue another strategy by offering precursors or derivatives that are transformed into plasmalogens after oral ingestion. One such option are alkylacylglycerols, which are closely related to the alkylglycerols described above but contain a pre-attached fatty acyl chain (in this case DHA) at sn-2. These compounds are metabolized to plasmalogens by introduction of a head group and the vinyl ether bond and *sn*-2 remodeling can occur in the tissues. Another company uses substances (PPI-1011, PPI-1025) with a pre-formed vinyl ether bond at *sn*-1, a fatty acyl chain (oleic acid or DHA) at *sn*-2 and a lipoyl group at *sn*-3 that increases stability and longevity. Also these precursors are converted into plasmalogens after oral intake and distributed across the body. Overall, the commercial enthusiasm about plasmalogens as nutritional or therapeutic supplements does not yet seem to match scientific evidence. Nevertheless, plasmalogen intake may have beneficial physiological effects under certain circumstances and involvement of these compounds or their derivatives in therapeutic strategies is a development to be monitored in the future.

### 4.2 Tissue-autonomous biosynthesis versus plasmalogen transport via the circulation

As outlined in chapter 2 *Steady state levels of plasmalogens in health and disease*, plasmalogen levels in the human body differ from tissue to tissue and are tightly regulated. How these tissue levels are reached, though, has not been shown. The liver has been proposed as the main location of plasmalogen synthesis with subsequent distribution via lipoproteins to the various tissues of the body (Snyder, 1972; Nagan and Zoeller, 2001; Braverman and Moser, 2012). Indeed, lipoproteins have high levels of plasmalogens and serum PE[P] levels reach 30% of total PE in rats and even 50% in humans. Different types of lipoproteins, including LDL, VLDL and HDL, all carry plasmalogens (Vance, 1990; Bräutigam et al., 1996) with highest levels reached in HDL (Ikuta et al., 2019). Regarding the role of plasmalogens in lipoproteins, there are reports showing them to be important for the protection from oxidative stress (Vance, 1990; Engelmann et al., 1994; Hahnel et al., 1999) with LDL and VLDL plasmalogens being more efficiently oxidized than HDL plasmalogens (Felde and Spiteller, 1995). Another role for ether lipids was shown in cellular models of total ether lipid deficiency, where they were found to impact on reverse cholesterol transport via HDL but the underlying mechanism is not clear (Munn et al., 2003).

From these findings it clearly emerges that plasmalogens are abundantly present in lipoproteins and may fulfill important roles there. Whether their presence in lipoproteins is also causally linked to their arrival in all tissues is not clear so far. Restoration of plasmalogen content after alkylglycerol treatment in rodents and humans, however, supports the conclusion that lipoproteins or also serum albumin are capable of transporting ether lipids via the blood.

There are, however, also a couple of findings that are in sharp contrast to the hypothesis of tissue distribution of plasmalogens by lipoproteins from the liver to the periphery. First of all, plasmalogen levels in the liver are very low (Braverman and Moser, 2012) and this is presumably due to the fact that FAR1, the rate-limiting enzyme in plasmalogen biosynthesis, is hardly active in liver and liver cell lines (Lee et al., 1980). Also transport of relevant amounts of plasmalogens across the blood brain barrier has never been achieved in feeding or supplementation experiments using ether lipid-deficient or wild type rodents (Das and Hajra, 1988; Das et al., 1992; Brites et al., 2011) and there are already indications that brain plasmalogens are produced locally rather than imported from outside (Honsho and Fujiki, 2017). In a study using a mouse model with hepatocyte-specific deletion of Pex5 (Dirkx et al., 2005), the peroxisomal targeting signal 1 receptor, which is essential for assembly of functional peroxisomes (Dodt et al., 1995), we found that plasmalogen levels in seven tissues and plasma were not affected by the inability of hepatocytes to synthesize ether lipids (Werner et al., submitted).

## 5 Conclusion

The interest in plasmalogens has risen continuously since their discovery in the 1920s. In the last years, plasmalogens have not only drawn the attention of experts in the field of lipid research but also of a broader audience involving specialists from various disciplines. This is on the one hand due to rapid advancements in lipid analysis allowing the determination of plasmalogen subspecies in a wide variety of organisms, tissues and cell types and on the other hand, to recent discoveries linking plasmalogens to important cellular pathways like ferroptosis or indicating their involvement in common diseases, for example Alzheimer’s disease or diabetes. However, to assess the role of plasmalogens in fundamental processes like ferroptosis or the etiology of complex diseases, it is imperative to understand in detail their physiological properties and regulation. Recent research has made big steps towards this goal, for example by the discovery of *TMEM189* as the gene coding for PEDS1 introducing the characteristic vinyl ether bond; or the revelation that strict homeostasis of plasmalogen levels is physiologically essential with both too high and too low levels causing similar and dramatic disease symptoms in humans. Nevertheless, several important aspects of plasmalogen biology remain in the dark. This is especially true for all facets of intra- and extracellular transport. Currently, it is still unclear, how ether lipid precursors are shuttled out of the peroxisome and, subsequently, to the ER for the last steps of plasmalogen biosynthesis. Similarly, little is known about plasmalogen transport between different tissues and here particularly, if and how plasmalogens can travel across the blood-brain barrier. Consequently, there is still a lot to learn about plasmalogens and their involvement in physiological and pathological processes and we are excited about what the future holds for research on these fascinating lipids.

## Abbreviations

AGMO, alkylglycerol monooxygenase; AGPS, alkylglyceronephosphate synthase; CDP-[E], CDP-ethanolamine; DG[O], 1-*O*-alkyl-2-acyl-*sn*-glycerol; DG[P], 1-*O*-alkenyl-2-acyl-*sn*-glycerol; DMA, dimethylacetal; ER, endoplasmic reticulum; FAR, fatty acyl-CoA reductase; GNPAT, glyceronephosphate *O*-acyltransferase; LPA[O], alkyl lysophosphatidic acid; LPA[P], alkenyl lysophosphatidic acid; LPE[P], alkenyl lysophosphatidylethanolamine; MG[O], monoalkylglycerol; PAF, platelet-activating factor; PA[O], alkyl phosphatidic acid; PBD, peroxisome biogenesis disorders; PEDS1, plasmanylethanolamine desaturase; PC, phosphatidylcholine; PC[O], plasmanylcholine; PC[P], plasmenylcholine (choline plasmalogen); PE, phosphatidylethanolamine; PE[O], plasmanylethanolamine; PE[P], plasmenylethanolamine (ethanolamine plasmalogen); PEX, peroxin; PL, phospholipase; PUFA, polyunsaturated fatty acid; RCDP, rhizomelic chondrodysplasia punctata.
